# Monosodium Urate Crystals Promote Innate Anti-Mycobacterial Immunity and Improve BCG Efficacy as a Vaccine against Tuberculosis

**DOI:** 10.1371/journal.pone.0127279

**Published:** 2015-05-29

**Authors:** Francesco Taus, Marilina B. Santucci, Emanuela Greco, Matteo Morandi, Ivana Palucci, Sabrina Mariotti, Noemi Poerio, Roberto Nisini, Giovanni Delogu, Maurizio Fraziano

**Affiliations:** 1 Department of Biology, University of Rome “Tor Vergata”, Rome, Italy; 2 Institute of Microbiology, Catholic University of Sacred Hearth, Rome, Italy; 3 Department of Infectious, Parasitic and Immuno-mediated Diseases, Istituto Superiore di Sanità, Rome, Italy; University of Cape Town, SOUTH AFRICA

## Abstract

A safer and more effective anti-Tuberculosis vaccine is still an urgent need. We probed the effects of monosodium urate crystals (MSU) on innate immunity to improve the Bacille Calmette-Guerin (BCG) vaccination. Results showed that *in vitro* MSU cause an enduring macrophage stimulation of the anti-mycobacterial response, measured as intracellular killing, ROS production and phagolysosome maturation. The contribution of MSU to anti-mycobacterial activity was also shown *in vivo*. Mice vaccinated in the presence of MSU showed a lower number of BCG in lymph nodes draining the vaccine inoculation site, in comparison to mice vaccinated without MSU. Lastly, we showed that MSU improved the efficacy of BCG vaccination in mice infected with *Mycobacterium tuberculosis* (MTB), measured in terms of lung and spleen MTB burden. These results demonstrate that the use of MSU as adjuvant may represent a novel strategy to enhance the efficacy of BCG vaccination.

## Introduction

The most recent report from WHO estimates 8.8 million new incident Tuberculosis (TB) cases worldwide and 1.3 million deaths [1], making TB one of the most deadly human infectious disease. The only currently licensed vaccine for TB is the Bacille Calmette-Guerin (BCG), an attenuated strain of *M*. *bovis*, which confers acceptable efficacy against disseminated forms of childhood TB, but highly variable and largely inadequate protection against the much more prevalent adult pulmonary TB [2]. Moreover, the fact that high proportions of HIV infected individuals live in TB endemic regions poses a significant risk when it comes to BCG (or other live) vaccine, given that the majority of serious complications following BCG administration occur in immunocompromised patients [3]. In this context, the generation of a safer and more efficacious BCG-based anti-TB vaccine may require the association of BCG with novel immunomodulators or adjuvants aimed at improving its intracellular killing by host phagocytes without affecting its efficacy as a vaccine.

Endogenous adjuvants are molecules released following tissue damage and have the capability to activate immune response by acting as danger associated molecular patterns (DAMPs) [4]. Among these molecules, monosodium urate crystals (MSU) are released from locally damaged cells and provide an adjuvant signal that alerts the immune system to danger [5]. Uric acid is a natural product of the catabolism of the purines that can generate crystals, and form precipitates of its sodium salt when it reaches supersaturation levels in body fluids [6]. In this context, deposition of MSU in joints can cause gout, an inflammatory disease characterized by joint swelling, due to infiltration of neutrophils [7]. Moreover, MSU may promote reactive oxygen species (ROS) generation [8] and NADPH oxidase (NOX) dependent neutrophil extracellular trap formation [9] as well as the release of proinflammatory cytokines [10]. Finally, MSU mediates the immunostimulatory effects of alum adjuvants by promoting inflammatory dendritic cell activation and antigen specific CD4 T [11] and CD8+ T [5] cell response, suggesting its potential use as endogenous adjuvant for novel vaccine formulations. On these grounds, the present study reports evidences showing the capability of MSU to induce *in vitro* a persisting innate anti-mycobacterial activation, which was associated *in vivo* with a better efficacy of BCG vaccination.

## Materials and Methods

### Bacteria and cell cultures

BCG Pasteur (TMC1011) and *Mycobacterium tuberculosis* (MTB) Erdman (TMC107) were grown and titred, as described [12]. Human pro-monocytic THP-1 leukemia cell line was grown as described [12], and induced to differentiate by 72 hour stimulation with 20 ng/ml Phorbol 12-Myristate 13-Acetate (PMA).

### Infection and quantification of mycobacteria

Differentiated THP-1 (dTHP-1) cells, used at the density of 5x10^5^/well, were exposed for 3 hours to BCG at the MOI (multiplicity of infection) of 1 in 24 well plates. After removal of extra-cellular bacilli, cells were stimulated or not with MSU (Enzo Life Sciences Inc.) at the concentration of 0.5, 5, 50 μg/ml and colony forming unit (CFU) assays were performed at day 3 and 5 post-infection, as previously described [12]. In several experiments, any possible effects, induced by MSU on phagocytosis, were evaluated by CFU assay performed on dTHP-1 cells after 3-hour exposure with BCG, administrated at the MOI of 1 in the presence or absence of 0.05, 0.5, 5 μg/ml MSU. In order to ascertain whether phagolysosome maturation and ROS generation were responsible for intracellular mycobacterial killing, 10 μM chloroquine, 20 mM NH_4_Cl or 100 U/ml poly-ethileneglycol (PEG)-catalase were added to BCG infected cells together with 5 μM MSU, as described [12].

In order to assess whether MSU could induce a persisting state of activation defined as “trained immunity” [13], non-differentiated dividing THP-1 cells, used as a model of human monocytes, were cultured with or without 5 or 50 μg/ml MSU for 3 days and then washed to remove the MSU stimulus. After further 4 days of culture, the cells were infected with BCG at the MOI of 10 for 3 hours. Thereafter, extracellular bacilli were removed by washing (T0) and cells were cultured for further 3 days (T3), in the presence or absence of 10 μM chloroquine. Intracellular mycobacteria were enumerated by CFU assay, as described [12].

Finally, in order to exclude any possible interference of MSU crystals with BCG viability, CFU assay was performed in BCG suspended in 200 μl of PBS containing or not 200 μg MSU crystals after 24 and 48 hours incubation at 37°C.

### Fluorimetric analysis

Intracellular Ca^2+^ was measured after labeling cells with the 3 μM fluorescent intracellular Ca^2+^ indicator Fluo-3/AM (Molecular Probes, NL), as described [12], followed by incubation at 37°C with 5 μg/ml MSU, for the times indicated in the figures. In several experiments, 20 μM BAPTA-AM (Sigma, MO), 3 mM ethylene glycol tetra-acetic acid (EGTA, Calbiochem, San Diego, CA) were added 30 minutes and 15 minutes before MSU addition, respectively. Intracellular Ca^2+^ was determined by fluorimetric analysis using 406 nm for excitation and 526 nm for emission.

ROS generation was analysed by loading cells with 10 μM of the fluorescent indicator 20,70-dichlorofluorescein diacetate (DCF, Molecular Probe) for 60 min at 37°C in the dark. Thereafter, cells were washed twice, stimulated for 20 minutes with 5 μg/ml MSU. ROS generation was determined by fluorimetric analysis using 488 nm for excitation and 530 nm for emission. In several experiments, in order to ascertain the role of Ca^++^ in ROS generation, either 20 μM BAPTA-AM (Sigma, MO) or 3 mM ethylene glycol tetra-acetic acid (EGTA, Calbiochem, San Diego, CA) were added 30 minutes or 15 minutes before MSU addition, respectively. As control, DCF labeled cells were treated with PEG-catalase 100U/ml for 30 minutes at 37°C and then stimulated for 20 minutes with 5 μg/ml MSU crystals.

Intraphagosomal acidification of vacuoles containing BCG was monitored by using BCG labelled with 100 μg/ml of the pH sensitive dye N-hydroxysuccin-imidyl 5-(and 6-)-carboxyfluorescein (NHS-CF) (Sigma), as described [14]; in particular, dTHP-1 cells were infected with NHS labelled BCG for 3 hours at the MOI of 5 and then stimulated overnight with 5 μg/ml of MSU. Intraphagosomal acidification was determined as a decrease of fluorescence intensity, measured at an excitation wavelength of 492 nm and emission wavelength of 517 nm.

Intracellular pH was determined in dTHP-1 cells infected with BCG for 3 hours at the MOI of 5, stimulated overnight with 5 μg/ml of MSU and then labelled with Lysosensor Green DN 189, as described [15]. Phagosomal acidification was determined by fluorescence intensity measured at an excitation wavelength of 443 nm and emission wavelength of 505 nm. The role of phagosomal maturation in the intraphagolysosomal pH was assessed by culturing cells in the presence of 10 μM chloroquine.

Proteolitic activity of dTHP-1 cells infected or not with BCG for 3 hours and stimulated or not overnight with 5 μg/ml of MSU was analysed by incubating cells with DQ-Red BSA (10 μg/ml, Molecular Probes) for 2 hours at 37°C. Proteolitic activity was then measured as fluorescence intensity measured at an excitation wavelength of 590 nm and emission wavelength of 620 nm.

All fluorimetric analysis were performed by the use of a Perkin Elmer LS50B luminescence spectrometer.

### Confocal microscopy analysis

The degree of maturation of endosomes containing BCG was assessed on BCG infected dTHP-1 cells stimulated for 18 hours with 5 μg/ml MSU, by analyzing the co-localization of bacilli with lysosomes after staining the mycobacteria with auramine and the lysosomes with Alexa Fluor 647 anti-LAMP-3 monoclonal antibody (IgG1, clone MX-49.129.5, Santa Cruz Biotechnology, Inc.), as described [12].

### IL-1β determination

The levels of IL-1β production in the supernatant were measured by a human IL-1β ELISA kit (Life Technologies), used according to the manufacturer’s instruction.

### Mice vaccination and MTB challenge

C57BL/6 female mice (Harlan) fed commercial mouse chow and water *ad libitum* and kept under pathogen-free conditions and used in accordance with institutional guidelines in compliance with national and international law and policies. All animal experiments were authorized by the Ethical Committee of the Università Cattolica del Sacro Cuore (permit number: Q21 462(A.290b)/C.E.Sp.An./2009). All efforts were made to minimize suffering; all manipulations were performed under medetomidine/ketamine anesthesia and mice were sacrificed by cervical dislocation after anesthesia. Five mice per group (matched for sex and age between 8 and 10 weeks) were vaccinated with i) 10^6^ CFU of BCG suspended in 200 μl PBS, ii) 10^6^ BCG suspended in 200 μl PBS containing 200 μg MSU crystals, or iii) 200 μl PBS alone, via subcutaneous route. The amount of MSU used *in vivo* has been chosen on the basis of adjuvant activity reported in previous studies [16–17].

To assess BCG persistence *in vivo*, the draining lymph nodes were removed aseptically and homogenized in 5 ml of 0.05% Tween80-PBS by using Seward Stomacher 80 blender (Tekmar, Cincinnati, OH). The homogenates were diluted serially in the Tween-80-PBS solution, and 50 μl aliquots were plated on Milddlebrook 7H11 agar (Difco, Detroit, MI). The number of CFU was determined after 20 days of incubation at 37°C in sealed plastic bags.

Ten weeks following immunization, vaccinated and control mice were infected aerogenically with ≈ 100 CFU/mouse of *M*. *tuberculosis* Erdman by using a Milddlebrook chamber (Glas-Col, Terre Haute, IN), as previously described [18]. The vaccinated and control mice were sacrificed 28 days after challenge, and bacterial colonization of lung and spleen tissues was assessed, as described [18].

### Statistical analysis

Statistical analysis was carried out by Graphpad Prism 3.0 software package. Comparison between groups was done using Student’s *t* test. p < 0.05 was considered statistically significant.

## Results

### MSU promote innate anti-mycobacterial response in human macrophages

**Fig 1:**
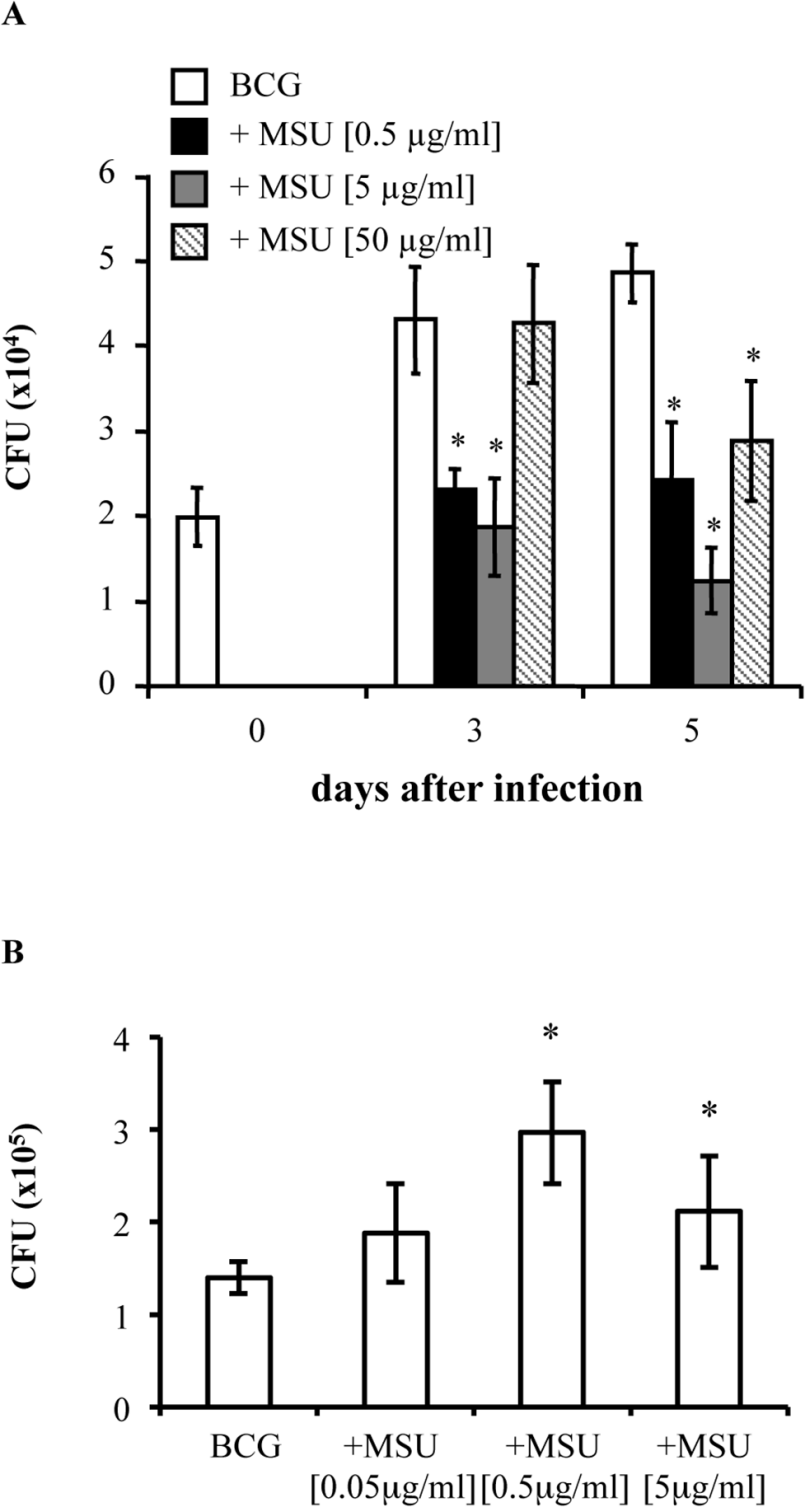
MSU crystals enhance antimycobacterial activity. (**A**) Differentiated THP-1 (dTHP1) cells were infected with BCG at the MOI of 1 and then stimulated or not with 0.5, 5, 50 μg/ml of MSU for 3 and 5 days. The results are expressed as means ± Standard Deviation (SD) of CFU values performed in triplicate and are representative of three independent experiments. * p ≤ 0.001 in comparison with non-stimulated control cells. (**B**) Stimulation of human macrophages with MSU enhances phagocytosis of BCG. Differentiated THP-1 cells were exposed to BCG at the MOI of 1 for 3 hour in the presence or not of 0.05, 0.5, 5 μg/ml MSU. Results are expressed as mean ± SD of CFU values performed in triplicate and are representative of two independent experiments. * p < 0.05 in comparison with non-stimulated control cells.

The pro-inflammatory effect of MSU [6], their capability to promote antigen presentation [11] and to drive T cell differentiation and polarization [19] have been previously documented, but data suggesting their possible effects in activating anti-mycobacterial innate immunity are not available. In this context, dTHP-1 cells infected with BCG at the MOI of 1 were stimulated with MSU and intracellular mycobacterial viability was evaluated by CFU assay. Results show a significant reduction of intracellular BCG colonies, at day 5 after infection, at all doses of MSU used, with a peak in the reduction observed with 5 μg/ml of MSU (Fig 1A). In addition, when the infection was performed in the presence of 0.5 and 5 μg/ml MSU we showed a significant increase of BCG phagocytosis measured after 3 hours from mycobacterial exposure (Fig 1B).

### MSU induce Ca^2+^ mediated ROS generation

**Fig 2:**
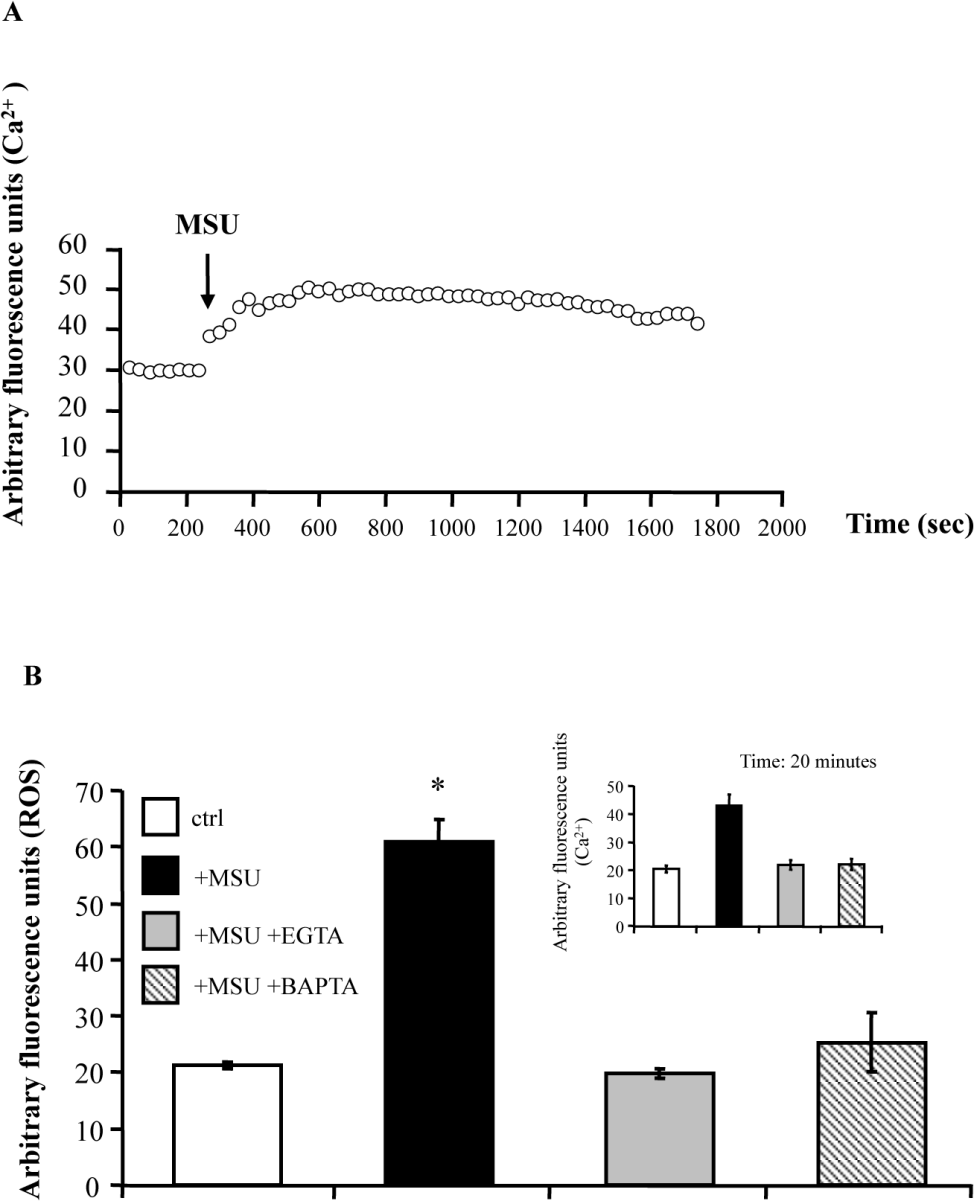
MSU crystals enhance Ca^2+^ dependent ROS production. (**A**) dTHP-1 cells were incubated with 3 μM Fluo-3/AM at 37°C for 1 hour in the dark and were stimulated with 5 μg/ml MSU. After stimulation, fluorescence emission was continuously monitored for 30 minutes and expressed as to determine relative alteration in intensity. (**B**) dTHP-1 cells were incubated for 1 hour at the dark with 10 μM DCF, or with 3 μM Fluo-3/AM (inset of the figure), and were stimulated with 5 μg/ml MSU. Ca^**2+**^ dependence ROS generation was assessed by adding 20 μM BAPTA-AM or 3 mM EGTA 30 minutes and 15 minutes before MSU addition, respectively. Fluorescence emission was monitored at 20 minutes after stimulation. Results are expressed as mean ± SD of arbitrary fluorescence units performed in triplicate and are representative of three separate experiments. * p < 0.01 in comparison with non-stimulated control cells

Intracellular calcium increase is required for many different signal transduction pathways, including activation of anti-mycobacterial responses [20, 21]. We therefore analyzed cytosolic Ca^2+^ influx in dTHP-1 cells following stimulation with MSU. Results show that cytosolic Ca^2+^ concentration peaks 10 minutes after MSU stimulation and remains almost constant up to the later time points (Fig 2A). The addition to the culture of EGTA and BAPTA-AM, an extracellular and intracellular Ca^2+^ chelator, respectively, caused a complete abrogation of cytosolic Ca^2+^ influx (inset of Fig 2B). Moreover, as Ca^2+^ is required for the assembly and activation of the superoxide-generating NADPH oxidase complex [22], we tested ROS generation and its possible dependence by Ca^2+^. Results show that MSU induce production of ROS, which are almost abrogated in the presence of EGTA and BAPTA-AM (Fig 2B), and in the presence of catalase (S1 Fig).

### MSU promote phagolysosome maturation

**Fig 3:**
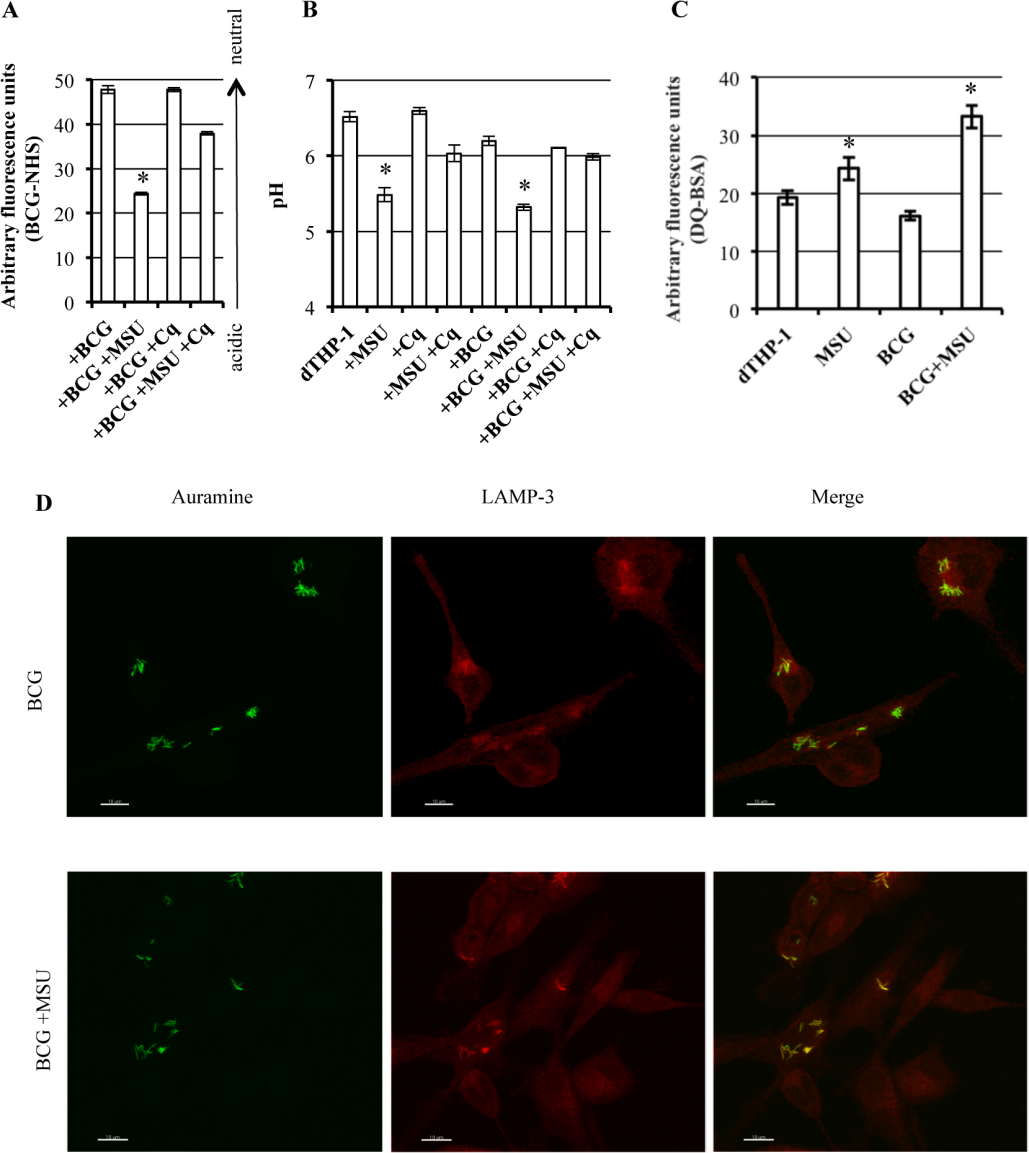
MSU crystals promote maturation of phagosomes containing BCG. (**A**) dTHP-1 cells were infected with NHS labelled BCG at the MOI of 1 and then stimulated overnight with 5 μg/ml of MSU, in the presence or absence 10 μM Chloroquine. Results are expressed in terms of mean ± SD of arbitrary fluorescence units of triplicate values and are representative of two independent experiments. * p < 0.001 in comparison with non-stimulated control cells, ° p < 0.001 in comparison with MSU stimulated cells. (**B**) dTHP-1 cells were infected with BCG, stimulated overnight with 5 μg/ml of MSU, in the presence or absence of 10 μM Chloroquine (Cq), and then labelled with 1 μM Lysosensor green DND 189. Results are expressed as mean ± SD of pH values from cultures performed in triplicate and are representative of two independent experiments. * p < 0.001 in comparison with non-stimulated control cells, ° p < 0.001 in comparison with BCG infected MSU stimulated cells (**C**) Protease activity of BCG infected dTHP-1 cells stimulated overnight with 5 μg/ml of MSU was analysed by loading of 10 μg/ml DQ red BSA for 2h at 37°C. Results are expressed as mean ± SD of triplicate values and are representative of three independent experiments. * p ≤ 0.01 in comparison with non-stimulated control cells. (**D**) Confocal microscopy representative images out of 10 per condition showing the increase of Auramine-stained BCG (green) residing in LAMP-3 positive vacuoles (red) after stimulation with 5 μg/ml of MSU. One representative experiment out of three is shown. (**E**) Summary of the mean percentage ± standard deviation (SD) of BCG co-localizing in LAMP-3-positive vacuoles determined by acquiring at least 10 images per condition and by counting ≥ 50 dTHP-1 cells per sample, after stimulation or not with MSU. Three different experiments were assessed. * p < 0.05 in comparison with BCG-infected cells (data were analyzed using the unpaired Student’s *t*-test).

To evaluate the maturation status of the phagosomes, we monitored phagosomal acidification by labelling BCG with *N*-hydroxysuccinimide (NHS)-carboxyfluorescein, a pH sensitive fluorochrome [15]. Results show that MSU promote acidification of phagosomes containing BCG and that this effect was significantly reverted, even if not completely, by the addition of choloroquine (Fig 3A), a lysosomotropic compound inhibiting phagolysosome acidification [12]. A quantitative analysis of intracellular pH demonstrated that MSU reduce intracellular pH both in BCG uninfected and infected macrophages and this effect was almost completely reverted in BCG infected macrophages by the addition of chloroquine, suggesting a phagolysosome driven process of intracellular acidification (Fig 3B). As phagolysosome acidification and maturation is associated with proteolitic enzyme activation, we monitored phagosomal protease activation by DQ-BSA staining [12]. Results show that MSU promote phagosome maturation and proteolysis activation in both BCG infected and uninfected macrophages (Fig 3C). Phagolysosome maturation was also analyzed by confocal microscopy in terms of co-localization of BCG with LAMP-3^+^ vacuoles. Results show that the stimulation with MSU significantly increased the co-localization of BCG with LAMP-3^+^ compartments in comparison to un-stimulated BCG infected control cells (Fig 3D and 3E).

### MSU reduce intracellular BCG growth by a phagolysosome mediated and ROS dependent mechanism

**Fig 4:**
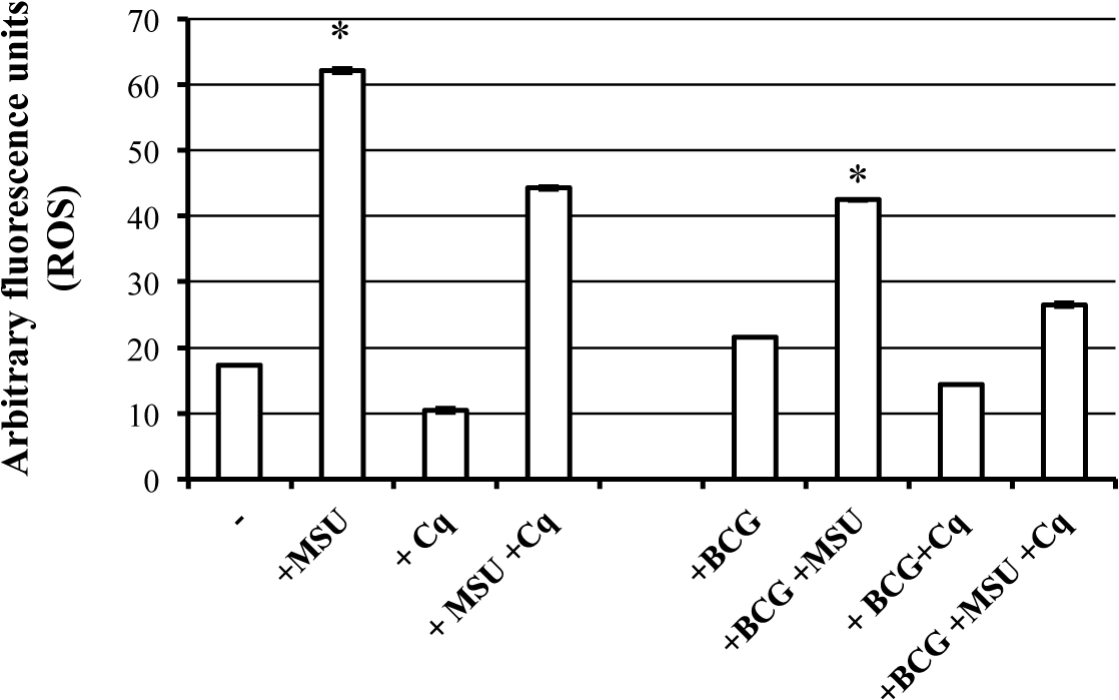
MSU crystals induce phagolysosome dependent ROS generation. dTHP-1 cells were infected or not with BCG at the MOI of 5 and then labelled with 10 μM DCF for 60 min. Thereafter, cells were washed twice, stimulated or not overnight with 5 μg/ml MSU in the presence or absence of 10 μM Chloroquine (Cq). Results are expressed as means ± SD of arbitrary fluorescence units of triplicate values and are representative of two independent experiments. * p < 0.001 in comparison with non-stimulated control cells. ° p < 0.001 in comparison with MSU stimulated cells or with MSU-stimulated BCG-infected cells.

Phagocytes generate ROS by using superoxide-generating NADPH oxidase (NOX) family proteins, which plays pivotal roles in host defence against bacterial and fungal pathogens [23], and whose polymerization occurs on maturing phagosomes [24]. Following overnight stimulation with MSU, a significant increase in ROS production was observed in both BCG infected and uninfected dTHP-1 cells in comparison with un-stimulated cells (Fig 4). Moreover, chloroquine addition inhibited 76% and 40% of ROS generation induced by MSU in BCG-infected and-uninfected dTHP-1 cells (p < 0.001), respectively, indicating that most of ROS production is of phagolysosomal origin during BCG infection.

**Fig 5:**
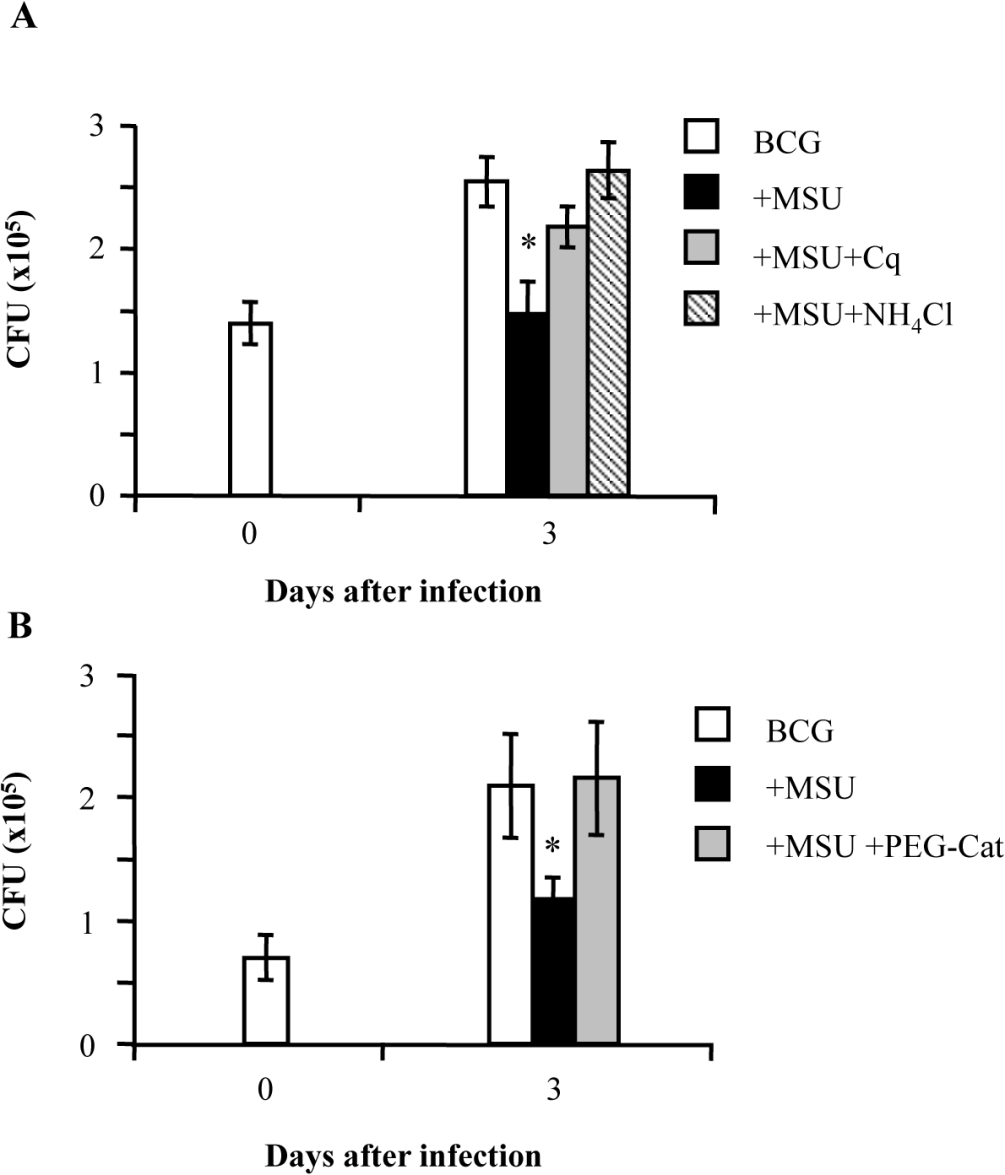
MSU crystals reduce intracellular BCG viability in a phagolysosome maturation dependent and ROS mediated manner. BCG infected dTHP-1 cells were stimulated or not with 5 μg/ml MSU and CFU assays were performed at day 3 post-infection. (**A**) In order to ascertain whether phagolysosome maturation was responsible for intracellular mycobacterial killing, 10 μM chloroquine (Cq) or 20 mM NH_4_Cl was added to BCG-infected cells together with MSU. Results are expressed as mean ± SD of CFU values performed in triplicate and are representative of two independent experiments. * p < 0.001 in comparison with non-stimulated control cells. (**B**) In order to ascertain the role of ROS in intracellular mycobacterial killing, 100 U/ml PEG-catalase was added to BCG infected cells together with MSU. Results are expressed as mean ± SD of CFU values performed in triplicate and are representative of two independent experiments. * p < 0.001 in comparison with non-stimulated control cells.

In order to assess the role of phagolysosome maturation and ROS production in intracellular mycobacterial killing induced by MSU, dTHP-1 cells were infected with BCG, stimulated with MSU and then exposed to the lysosomotropic agents chloroquine or NH_4_Cl which both increase intralysosomal pH and are considered general lysosomal inhibitors. Results show that the anti-mycobacterial activity increased by MSU was mediated by phagolysosome maturation and acidification since the addition of chloroquine or NH_4_Cl upon MSU treatment significantly increased intracellular mycobacterial viability to values closed to control cells (Fig 5A). Moreover, to assess the role of ROS in the MSU induced intracellular mycobacterial killing, BCG-infected cells were exposed to PEG-Catalase (PEG-Cat). The results indicate that PEG-Cat abolishes MSU crystal induced intracellular mycobacterial killing (Fig 5B).

### MSU induce trained anti-mycobacterial innate immunity

**Fig 6:**
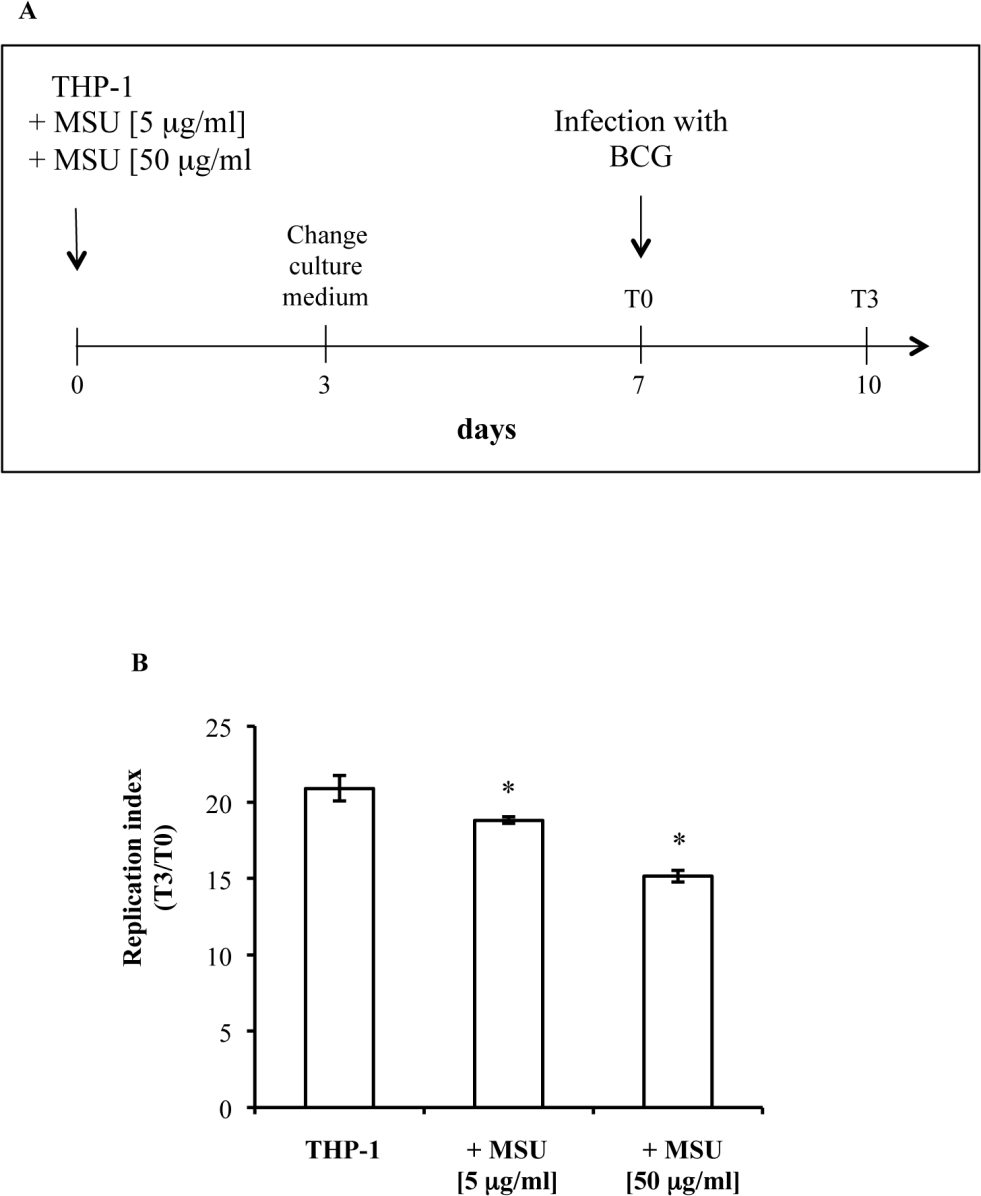
MSU crystals induce trained anti-mycobacterial innate immunity. (**A**) Diagram showing the course of *in vitro* preincubation experiment. THP-1 cells were cultured with 5 or 50 μg/ml MSU for 3 days. Thereafter, the medium was changed to remove MSU stimulus and cells cultured for further 4 days. Finally, cells were exposed to BCG at the MOI of 10 for 3 hours (T0), washed and cultured for further 3 days (T3). (**B**) Intracellular mycobacterial growth was monitored in BCG infected THP-1 cells prestimulated or not with 5 or 50 μg/ml MSU. Results are shown as mean ± SD of CFU values performed in triplicate and are representative of three independent experiments. ° p < 0.05 and * p < 0.001 in comparison with same time non pre-stimulated control cells. (**C**) Intracellular mycobacterial growth was monitored in THP-1 cells, pre-stimulated or not with 50 μg/ml MSU, and exposed or not to 10 μM chloroquine after BCG infection for 3 days. Results are shown as mean ° SD of CFU values performed in triplicate. * p < 0.05 and ° p < 0.01 in comparison with same time non pre-stimulated control cells. (**D**) IL-1β production in the supernatant of BCG infected THP-1 cells pre-stimulated or not with either 5 or 50 μg/ml MSU. Results are shown as mean ± SD of values performed in triplicate and are representative of three independent experiments. * p < 0.05 in comparison with same time non pre-stimulated control cells.

A functional reprogramming of monocytes leading to a trained innate immunity has been recently reported following infection with *Candida albicans* or stimulation with TLR-4 ligands such as β-glucan [13]. On these ground, we wondered whether the antimicrobial activity induced by MSU could persist over time following stimulation of non differentiated, dividing THP-1 cells, used as a model of human monocytes. To this aim, THP-1 cells were stimulated according to the protocol shown in Fig 6A and infected 7 days after MSU stimulation. Intracellular mycobacterial growth was evaluated in terms of CFU obtained immediately after 3 hour mycobacterial exposure (T0) and after 3 days of infection. Results show a significant dose-dependent decrease of intracellular mycobacterial growth following pre-stimulation of monocytes with MSU (Fig 6B), suggesting the capability of MSU to generate an enduring anti-mycobacterial activity. Moreover, treatment of BCG infected THP-1 pre-stimulated or not with MSU and then treated with chloroquine, a drug known to arrest phagolysosome maturation, caused higher mycobacterial CFU counts than unstimulated THP-1, suggesting that phagolysosome maturation is required for both MSU induced and basal intracellular mycobacterial killing (Fig 6C). Finally, the increase in antimycobacterial activity was associated with a significant increase of IL-1β production in cells pre-stimulated with either 5 or 50 μg/ml MSU.

### The co-administration of BCG and MSU enhances the efficacy of BCG vaccination in a mouse model

**Fig 7:**
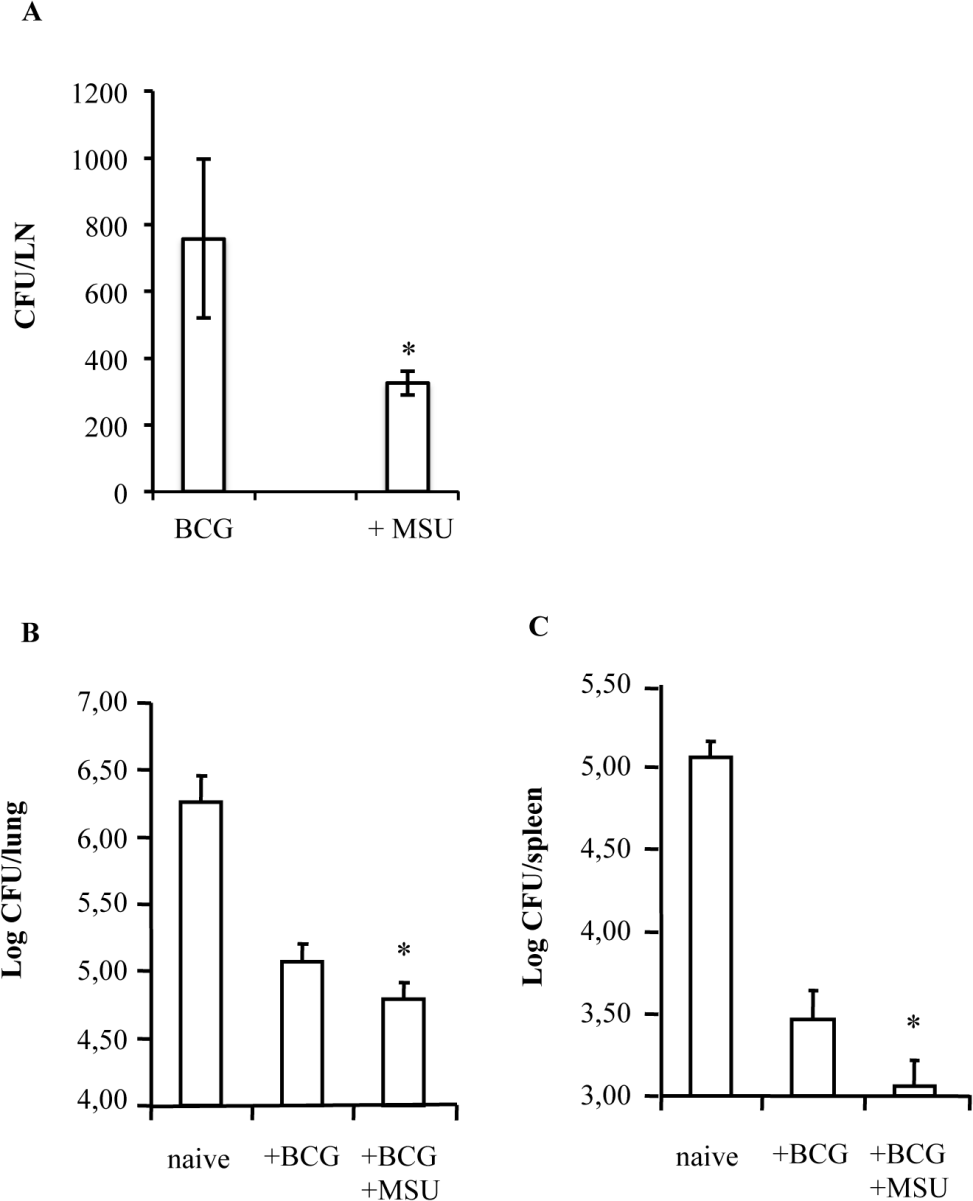
Co-administration of BCG with MSU crystals enhances the clearance and efficacy of BCG vaccination. Five mice per group were vaccinated with i) 10^**6**^ CFU BCG, ii) 10^**6**^ CFU BCG+MSU [200 μg], or iii) PBS alone. (**A**) Mice were sacrificed after 15 days from immunization and BCG colonies enumerated by the draining axillary lymph nodes. Immunized and control mice were infected 10 weeks post immunization with *M*. *tuberculosis* Erdman (≈ 100 CFU/mouse) by the aerogenic route. Twenty-eight days later, mice were sacrificed and bacterial loads were determined by CFU counting in the lungs (**B**) and spleens (**C**). * p < 0.05 in comparison with BCG vaccinated mice.

BCG is a live attenuated vaccine with debatable efficacy, but considered safe since rarely its administration is associated to severe adverse events. As a live attenuated vaccine, however, BCG is not recommended in immunocompromised patients, such as HIV+ individuals [25]. The capability of MSU to activate and train innate anti-mycobacterial response prompted us to test their possible use as adjuvants in a novel BCG-based vaccine formulation in order to increase both the efficacy of anti-TB vaccination and the clearance of BCG after inoculation in order to reduce the risks of BCGitis in immunocompromised patients. As a measure of clearance, in a first series of experiments, BCG was inoculated in mice in the presence or absence of MSU and 15 days later, mycobacterial CFU were enumerated in the lymph nodes draining the inoculation site. Results (Fig 7A) showed that the association of MSU to BCG causes a significant *in vivo* killing of BCG, measured as a significant reduction of BCG CFU in draining lymph nodes. In this context, any direct modification on BCG viability was not observed by the presence of MSU in the novel vaccine formulation (S2 Fig). To test the efficacy of BCG vaccination in association to MSU as adjuvants we used a mouse model of aerosolic infection with MTB [18]. Briefly, naïve mice or mice vaccinated with BCG or with BCG/MSU were aerogenically infected with MTB 10 weeks after the immunization. Mice were euthanized 4 weeks after infection and both lungs and spleens were removed for the analysis of mycobacterial burden. Results show that MTB colonies both in the lungs and in the spleens (Fig 7B and 7C, respectively) were significantly reduced in mice vaccinated with BCG and MSU in comparison to mice vaccinated with BCG alone.

## Discussion

BCG actually represents the sole anti-TB immuno-prophylactic tool available for vaccination of neonates and individuals at increased risk of MTB infection. It is a live vaccine with documented protecting activity against systemic forms of TB in childhood [26], but its protective effect against pulmonary disease in childhood is variable and there is no evidence for protective effects in HIV infected children [26]. Moreover, WHO stopped to recommending live, attenuated, BCG vaccination at birth for asymptomatic HIV infected infants, even where there is a risk of TB exposure early in life, due to high risk of disseminated BCG disease [26]. These evidences claim the need for a safer, and possibly more efficient, anti-TB vaccine, replacing or improving the already available BCG vaccine [27]. In this context, out of 14 anti-TB vaccine actually in clinical pipeline, 5 are indicated for HIV infected patients and consist mainly of viral vector or subunit vaccines [28–29]. In the present study, we have evaluated the possibility to improve the existing vaccine by associating BCG with MSU aiming at increasing its clearance after administration to reduce the risks of BCGitis and without reducing or even improving its efficacy.

Precipitation of MSU in individuals with hyperuricemia causes Gout symptomatology by inducing a strong acute inflammatory response in joints and bursal tissues [30]. Acute inflammatory reaction induced by MSU involves the activation of the inflammasome NLRP3 as well as IL-1β and IL-18 production [31], which in turn mediates the expansion of the pro-inflammatory T helper 17 cells [19]. In the present study, we have demonstrated on the one hand the inefficacy of MSU to directly affect BCG viability and, on the other hand, the capability of MSU to enhance phagocytosis and induce restriction of intracellular BCG growth, so adding to its intrinsic and well-known pro-inflammatory properties the capability to increase antimicrobial innate immune response. Unexpectedly, the effects in macrophages appeared to be inversely correlated to the dose of MSU used, with an effect, in terms of CFU reduction, higher at 5 μg/ml than at 50 μg/ml. These results can reflect different modes of interaction of MSU with cell membrane, depending by the dose used. In this context, low amounts of MSU may directly interact with CD14/TLR2/TLR4 systems to enhance microbicidal response, whereas higher amounts may induce lipid sorting on cell membrane to form more optimized interactions with solid surfaces, and allowing phagocytosis but not the activation of mycobacteriocidal signal transduction pathways [32].

Ca^2+^ is a ubiquitous second messenger that controls multiple processes in phagocytes, including the activation of microbicidal mechanisms and it is required for phagolysosome maturation [12] and the assembly and activation of superoxide–generating NADPH oxidase complex [22]. Intracellular Ca^2+^ elevation was observed in neutrophil following stimulation with inflammatory amounts of MSU [33]. Our results show that MSU induce cytosolic Ca^2+^ mobilization and promote Ca^2+^ mediated ROS generation also in human macrophages. NADPH oxidase assemblies on maturing phagosomes and ROS generation can be used as a measure of phagosomal maturation [22]. Moreover, both MTB and BCG are able to inhibit functional maturation and acidification of phagosomes by preventing fusion with acidic, hydrolytic lysosomes [34]. Our results show that MSU induce acidification and maturation of phagosomes containing BCG, as detected in terms of pH acidification and decrease of pH sensitive NHS fluorescence. In fact, the addition of the lysosomotropic agent chloroquine, which can be considered as general lysosomal inhibitor [12, 20], in combination with MSU to BCG-infected cells significantly increases the phagosomal pH, making it similar to that observed in the absence of MSU. Finally, these processes are necessary for ROS generation and intracellular mycobacterial killing, as the treatment with Chloroquine or NH4Cl (another general lysosomal inhibitor) [12,20], significantly reduces ROS and enhances intracellular mycobacterial viability.

A functional reprogramming of human monocytes leading to a form of innate memory has been recently described [35]. Monocytes treated with *Candida albicans* [13], BCG [36] or β-glucan [13] showed enhanced pro-inflammatory cytokine production (such as IL-1 β, TNF-α and IL-6), which persisted for 1 week after initial stimulation. This form of innate memory was associated to stable changes in histone trimethylation at H3K4, suggesting the involvement of epigenetic mechanisms in this phenomenon. Results reported herein show that pre-stimulation of human monocytes with MSU induced a persistent capability to restrict intracellular BCG growth, by a phagolysosome maturation dependent mechanism, maintained by cell progeny up to day 10 from stimulation. Moreover, the increased anti-mycobacterial response observed in MSU pre-stimulated cells was also associated with a significant increase of IL-1βsecretion, further suggesting that an epigenetic reprogramming of monocytes is operative in our experimental model. Furthermore, the superior protection, as anti-TB vaccine, induced by BCG ΔureC::hly over parental BCG has been described to be associated with inflammasome activation and autophagy [37] and IL-1β has been reported to confer host resistance through the induction of eicosanoids that limits excessive type 1 interferon production and foster mycobacterial containment [38]. On these grounds, immunization strategies targeting these innate immune pathways could represent additional tools for rational design of novel anti-TB vaccines.

The possibility that MSU could be used *in vivo* as novel endogenous adjuvants has been previously suggested in murine models of tumor immune rejection [39]. Moreover, the mechanism of action of the well-known adjuvant alum has been recently reported to be dependent, at least in part, by the local accumulation of MSU [11]. However, it remains a question as to why alum mediates only antibody responses whereas MSU stimulates a prevalent cell mediated immune response. In this context, future studies may reveal additional regulatory mechanisms unique to alum that suppress cytolitic T cell induction. Finally, the safety of intradermal injection of MSU in healthy human volunteers has been reported [40]. Although this latter study was not characterized as a phase 1 clinical trial, it represents a preliminary study showing apparent absence of MSU toxicity in humans and may provide the basis for future controlled clinical trials. Results reported herein show that MSU when administrated in mice in association to BCG, significantly reduce BCG colonies in draining lymph nodes, in agreement with their effects observed *in vitro*. Although the reduction of BCG viability is considered a contraindication for the maintenance of a long-lasting protective effect, it may be favored in conditions of high risk for HIV infection, provided that its efficacy profile is not affected. In this context, our results show that mice vaccinated with BCG and MSU show a significant improvement in lung and spleen MTB burden.

The absence of a clear and unequivocal correlate of protection identified by the T cell immune response against MTB [2] makes it difficult to identify a defined vaccine strategy against TB, which remains a severe disease both in immunocompetent and in immunocompromised individuals, such as HIV-infected subjects. In this context, the herein proposed novel formulation BCG and MSU may take advantage by the well-known adjuvant properties of MSU and by its capability, reported herein, to enhance a persisting anti-mycobacterial response by innate immune cells.

## Supporting Information

S1 Fig PEG-catalase pre-treatment reduces ROS levels induced by MSU. dTHP-1 cells were labeled with 10 μM DCF for 60 min. Thereafter, cells were washed twice, stimulated for 20 minutes with 5 μg/ml MSU in the presence or absence of 100 U/ml PEG-catalase (Cat). Results are expressed as means ± SD of arbitrary fluorescence units of triplicates and are representative of two independent experiments. * p < 0.05 in comparison with non stimulated control cells, **°** p < 0.05 in comparison with MSU stimulated cells.
(TIF)

S2 Fig Evaluation of BCG stability following formulation with MSU. BCG was suspended in 200 μl PBS in the presence or absence of 200 μg MSU crystals in order to mimic BCG formulation which was administrated in mice. Mycobacterial viability was monitored by CFU assay at 24 and 48 hours incubation at 37°C. Data are expressed as mean ± S.D. of CFU values performed in triplicate. n.s. = not significant in comparison with control BCG.
(TIF)
