# Supplementary figures and images for: Monosodium Urate Crystals Promote Innate Anti-Mycobacterial Immunity and Improve BCG Efficacy as a Vaccine against Tuberculosis
Source: PLoS One. 2015 May 29;10(5):e0127279. doi: 10.1371/journal.pone.0127279 (PMC4449037; doi:10.1371/journal.pone.0127279)

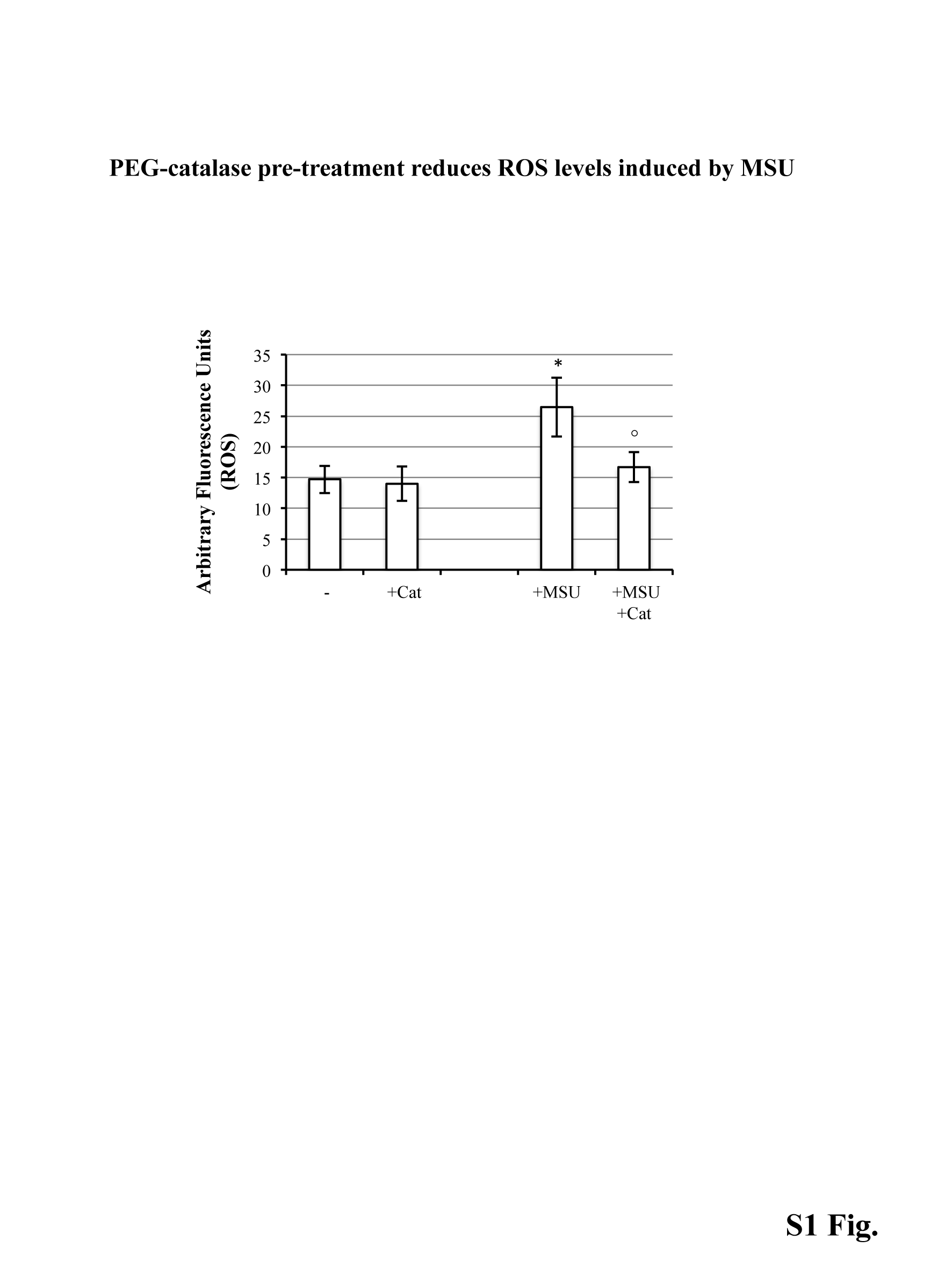

Supplement: S1 Fig — dTHP-1 cells were labeled with 10 μM DCF for 60 min. Thereafter, cells were washed twice, stimulated for 20 minutes with 5 μg/ml MSU in the presence or absence of 100 U/ml PEG-catalase (Cat). Results are expressed as means ± SD of arbitrary fluorescence units of triplicates and are representative of two independent experiments. * p < 0.05 in comparison with non stimulated control cells, ° p < 0.05 in comparison with MSU stimulated cells. (TIF) [file pone.0127279.s001.tif]

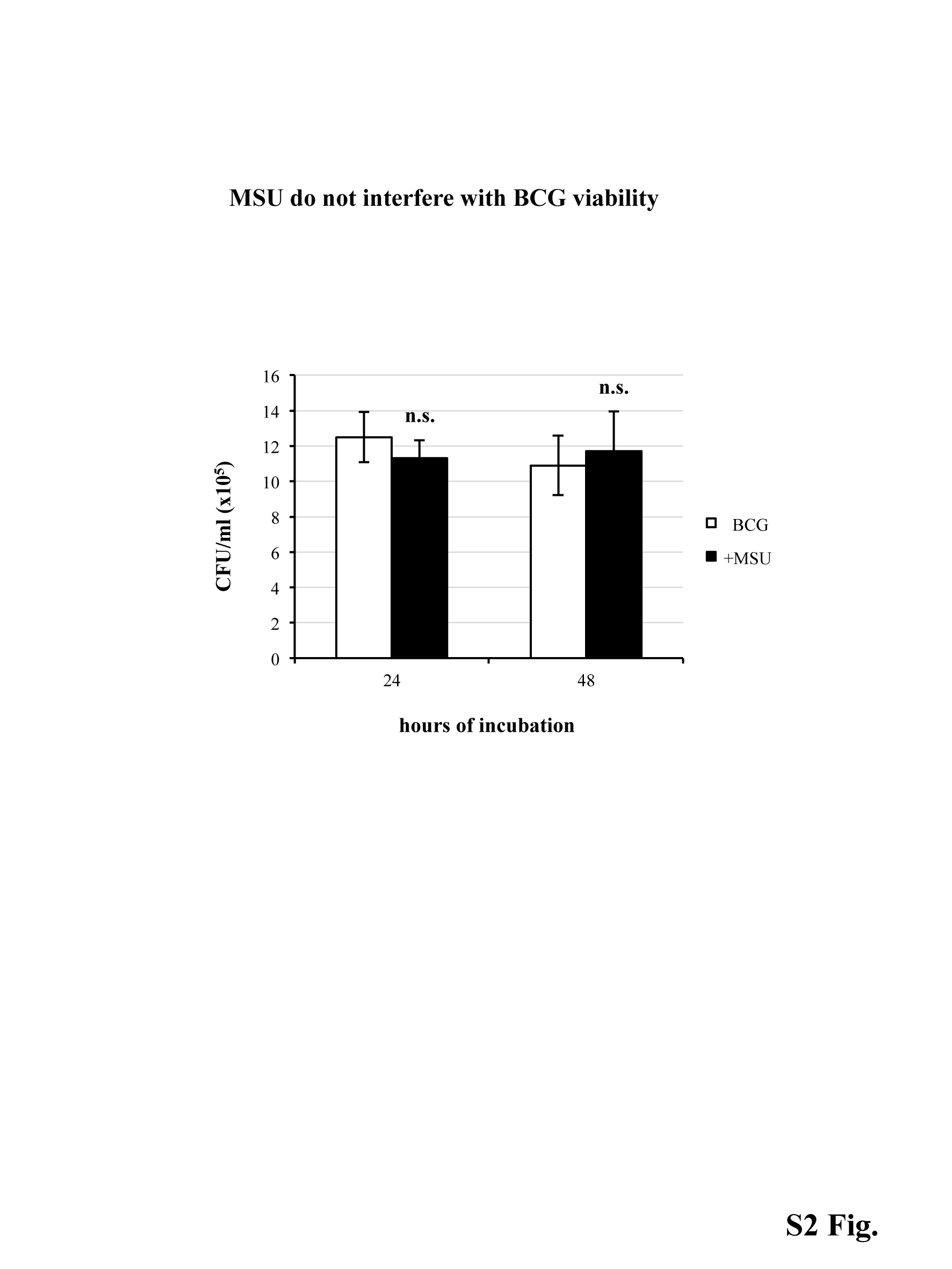

Supplement: S2 Fig — BCG was suspended in 200 μl PBS in the presence or absence of 200 μg MSU crystals in order to mimic BCG formulation which was administrated in mice. Mycobacterial viability was monitored by CFU assay at 24 and 48 hours incubation at 37°C. Data are expressed as mean ± S.D. of CFU values performed in triplicate. n.s. = not significant in comparison with control BCG. (TIF) [file pone.0127279.s002.tif]
